# Self-Microemulsifying Drug Delivery System to Enhance Oral Bioavailability of Berberine Hydrochloride in Rats

**DOI:** 10.3390/pharmaceutics16091116

**Published:** 2024-08-24

**Authors:** Xiaolan Chen, Haifeng Yang, Longyu Shi, Yujuan Mao, Lin Niu, Jing Wang, Haifeng Chen, Jiping Jia, Jingxuan Wang, Jiajie Xue, Yan Shen, Chunli Zheng, Yu Tian, Yi Zheng

**Affiliations:** 1 Department of Pharmaceutics, Jiangsu Agri-Animal Husbandry Vocational College, No. 8, Fenghuang East Road, Taizhou 225300, China; 2006010154@jsahvc.edu.cn (X.C.); 2006010159@jsahvc.edu.cn (H.Y.); 2004020062@jsahvc.edu.cn (Y.M.); 2003010168@jsahvc.edu.cn (L.N.); 2014010351@jsahvc.edu.cn (J.W.); 2006020069@jsahvc.edu.cn (H.C.); 2007030029@jsahvc.edu.cn (J.J.); 202214762@jsahvc.edu.cn (J.W.); 202210863@jsahvc.edu.cn (J.X.); 2 College of Life Sciences, China Pharmaceutical University, Nanjing 210009, China; 2020211682@stu.cpu.edu.cn; 3 Department of Pharmaceutics, China Pharmaceutical University, Nanjing 210009, China; shenyan@cpu.edu.cn (Y.S.); 020041357@cpu.edu.cn (C.Z.); 4 School of Medicine, Shanghai University, Shanghai 200444, China; 5 Institute of Geriatrics (Shanghai University), Affiliated Nantong Hospital of Shanghai University (The Sixth People’s Hospital of Nantong), School of Medicine, Shanghai University, Nantong 201613, China

**Keywords:** SMEDDS, berberine hydrochloride, bioavailability, in vitro dissolution

## Abstract

Berberine hydrochloride (BH) is a versatile bioactive compound derived from the plants of the *Berberis* genus, known for its various pharmacological effects. However, its oral bioavailability is low due to its high hydrophilicity and limited permeability. To enhance its clinical efficacy and oral bioavailability, this study designed and prepared a BH-loaded self-microemulsifying drug delivery system (BH-SMEDDS), and characterized its in vitro and in vivo properties. Firstly, the optimal formulation of BH-SMEDDS was selected using solubility evaluations, pseudo-ternary phase diagrams, and particle size analysis. The formulation containing 55% Capmul MCM, 22.5% Kolliphor RH 40, and 22.5% 1,2-propanediol was developed. BH-SMEDDS exhibited stable physicochemical properties, with an average particle size of 47.2 ± 0.10 nm and a self-emulsification time of 26.02 ± 0.24 s. Moreover, in vitro dissolution studies showed significant improvements in BH release in simulated intestinal fluid, achieving 93.1 ± 2.3% release within 300 min. Meanwhile, BH-SMEDDS did not exhibit cytotoxic effects on the Caco-2 cells. Additionally, BH-SMEDDS achieved a 1.63-fold increase in oral bioavailability compared to commercial BH tablets. Therefore, SMEDDS presents a promising strategy for delivering BH with enhanced oral bioavailability, demonstrating significant potential for clinical application.

## 1. Introduction

Berberine is a natural quaternary ammonium isoquinoline alkaloid extracted and isolated from plants of the Berberis genus, such as *Coptis chinensis* (Chinese goldthread) and *Phellodendron amurense* (Amur cork tree) [1]. In its natural state, berberine is commonly found as a chloride salt with low water solubility, belonging to the alkaloid family of protoberberine (5,6-dihydrodibenzo[a,g]quinolizinium). Berberine hydrochloride (BH) is produced by reacting berberine with hydrochloric acid, enhancing its water solubility and thereby improving its suitability for pharmaceutical formulation and application [2,3]. BH has various pharmacological effects including antidiabetic, antitumor, anti-inflammatory, antimicrobial, and antiatherosclerotic activities [4,5,6,7,8,9]. For instance, BH is utilized in the treatment of type 2 diabetes by activating AMP-activated protein kinase, thereby enhancing insulin sensitivity and reducing insulin resistance [5]. BH also modulates gut microbiota composition and metabolism, leveraging its antimicrobial properties against gut pathogens to potentially benefit the treatment of diarrhea and other digestive issues [7,9].

Oral administration is the preferred route due to its convenience and better patient compliance. Currently, BH is available in marketed formulations such as tablets and capsules. However, the oral administration of BH has demonstrated low bioavailability, mainly due to its strong hydrophilicity from the quaternary ammonium group, and low cell membrane permeability [10,11]. Fortunately, various formulation strategies have been explored to enhance the solubility and bioavailability of BH. The research indicates that the use of nanotechnology, such as nanoemulsions, nanoparticles and nanocrystals, appears to be an effective delivery system for improving solubility, absorption and oral bioavailability of BH [11,12,13].

Self-microemulsifying drug delivery systems (SMEDDS) are an ideal delivery strategy for improving the oral delivery of hydrophobic drugs [14,15]. Besides enhancing the bioavailability, SMEDDS offer other benefits such as increased solubility and dissolution rate, improved membrane permeability in the gastrointestinal tract, high drug loading efficiency, and ease of preparation [16,17]. SMEDDS consist of a drug, oil phase, surfactant, and co-surfactant. On the one hand, hydrophobic drugs can be encapsulated in solubilized microemulsions in the gastrointestinal tract, thereby enhancing their dissolution rate [18,19]. On the other hand, SMEDDS automatically form an oil-in-water (O/W) microemulsion under the digestive movements of the stomach and intestines, creating numerous emulsion droplets with particle sizes less than 100 nm. This small-sized microemulsion provides a large interfacial area, promoting gastrointestinal permeability of the incorporated drug and improving its absorption [20,21]. Therefore, SMEDDS represent a promising delivery system for transporting BH in the gastrointestinal tract, enhancing its solubility, membrane permeability, drug absorption, and ultimately, its bioavailability.

In this study, the BH-loaded SMEDDS was developed. Based on the solubilizing ability and pseudo-ternary phase diagrams, suitable oils, surfactants, and co-surfactants to prepare BH-SMEDDS were selected. The formulation was optimized using particle size and size distribution of the emulsions. Furthermore, the optimal formulation of BH-SMEDDS was characterized according to transmission electron microscopy, emulsification duration, and stability assessments. Subsequently, in vitro drug release and cytotoxicity studies were conducted. Finally, an in vivo pharmacokinetic study of the BH-SMEDDS was evaluated in rats.

## 2. Materials and Methods

### 2.1. Reagents and Materials

Berberine hydrochloride was purchased from the Titan Technology Co., Ltd. (Shanghai, China, batch no. 0111043444). Berberine hydrochloride tablets were purchased from the Shanghai Xinyi Tianping Pharmaceutical Co. (Shanghai, China, batch no. 230302). Methanol, LC grade methanol, and acetonitrile were purchased from Starco High Purity Solvents Co. (Shanghai, China). Potassium dihydrogen phosphate dihydrate, OP-10, polyethylene glycol 400 (PEG 400), isopropyl alcohol, 1,2-propanediol, glycerol, and potassium dihydrogen phosphate dihydrate were purchased from Lingfeng Chemical Reagent Co. (Shanghai, China). Oleic acid, ethyl oleate, castor oil, and Tween 80 from Sinopharm Chemical Reagent Co. (Shanghai, China). Glyceryl monooleate (Peceol^TM^), medium chain triglycerides (MCT), glyceryl monolinoleate (Maisine CC), caprylic capric glycerol (Labrasol^®^), polyethylene glycerol laurate (Gelucire^®^ 44/14), diethylene glycol monoethyl ether (Transcutol^®^ HP), polyethylene glycerol oleate (Labrafil M1944 CS), propylene glycol monocaprylate (Capryol 90), and lauryl alcohol-90 (Lauraglycol-90) were purchased from Gaitefosse (Saint-Priest, France). Caprylic mono/diglyceride (Capmul^®^ MCM), propylene glycol mono octanoate (Capmul^®^ PG8), and medium chain triglycerides (Captex 300 EP/NF) were supplied by Abitec (Janesville, WI, USA). Polyoxyethylene hydrogenated castor oil (Kolliphor^®^ RH40) and polyoxyethylene 35 castor oil (Kolliphor^®^ EL) were purchased from BASF AG (Frankfurt am Main, Germany) Heparin sodium was obtained from Yuan Ye Biotechnology Co. (Shanghai, China). Palmatine hydrochloride was purchased from Solebrite Technology Co. (Beijing, China, batch no. 2642332). All other chemicals used were of analytic grade. The human colon cancer cell line Caco-2 (ATCC number: HTB37) was purchased from the American Tissue Culture Collection (ATCC, Manassas, VA, USA). 

### 2.2. Animals

Male Sprague Dawley (SD) rats weighing 200 ± 20 g were purchased from the Qinglongshan Laboratory Animal Company (Nanjing, China). All rats were housed under strictly controlled ambient temperature (23 ± 1 °C, RH 20%) with artificial illumination (a 12 h light on/off cycle). This study was approved by the Ethical Committee of the China Pharmaceutical University (20230510).

### 2.3. Determination of Solubility and Oil-Water Partition Coefficient (P_o/w_) of BH

The equilibrium solubility at various pH conditions and P_o/w_ in simulated gastric and intestinal fluid conditions were determined. To establish various pH conditions, solutions of hydrochloric acid at a pH of 1.2, a pH of 2.0, a pH of 3.0, acetate buffer at pH 4.0, and phosphate buffer at a pH of 5.0 and a pH of 6.8 were prepared following pharmacopoeia guidelines. An excess amount of BH was added to each cap vial containing the corresponding solution. The suspension was thoroughly mixed and maintained at 100 rpm and 37 °C for 48 h until reaching equilibrium. Subsequently, the mixture underwent centrifugation at 8000 rpm for 30 min, followed by methanol dilution for quantitative analysis using a UV spectrophotometer (UV-1800, Shanghai Jinghua Instrument Ltd., Shanghai, China) at 350 nm.

The P_o/w_ of BH at different pH conditions was assessed by the shake-flask method [22]. A precise amount of 20 mg of BH was weighed and dissolved in water-saturated *n*-octanol to obtain a 200 μg/mL of BH *n*-octanol solution. Subsequently, 8 mL of *n*-octanol saturated solutions with pH values of 1.2 or 6.8 were mixed with 1 mL of BH *n*-octanol solution. These mixtures were further shaken with a speed of 100 rpm at 37 °C for 48 h to obtain equilibrium. After centrifugation at 8000 rpm for 20 min, the water phase and *n*-octanol phase of samples were separated, and the drug concentrations in each phase (Co for *n*-octanol phase and C_w_ for aqueous phase) were promptly determined using a UV spectrophotometer at 350 nm. The P_o/w_ was calculated according to Formula (1), as follows:

(1) $$P_{o/w}=\frac{C_{0}V_{W}}{C_{W}V_{0}}$$

where C_w_ and C_0_ represent the BH concentration in aqueous phase and *n*-octanol phase, respectively, at equilibrium; meanwhile, V_w_ and V_0_ represent the volume of aqueous phase and *n*-octanol phase, respectively.

### 2.4. Development of BH-SMEDDS Formulation

#### 2.4.1. Determination of the Solubility of BH in Various Excipients 

The equilibrium solubility of BH in various surfactants, co-surfactants, and oils was determined. The excess amount of BH was added to different oils (oleic acid, ethyl oleate, castor oil, Captex 300 EP/NF, Peceol, MCT, Maisine™ 35-1, Capmul MCM, Capmul PG 8, Labrafil M 1944 CS, Capryol 90, Lauraglycol-90), surfactant (Tween 80, Kolliphor RH 40, Kolliphor EL, Labrasol, Gelucire 44/14, and OP-10), and co-surfactant (Transcutol HP, PEG 400, isopropanol, propanetriol, and 1,2-propanediol). The mixture was subsequently vortexed and gently shaken at 100 rpm/37 °C (LY20-211C Constant Temperature Shaker, Longyue Instrument Co., Ltd., Shanghai, China) for 48 h until equilibrium was reached. Then, the mixture was centrifuged at 8000 rpm for 30 min (H1750R High Speed Freezer Centrifuge, Beckman Coulter Co., Ltd., Brea, CA, USA), and the supernatant was diluted with methanol. The absorbance of BH was measured at 350 nm using a UV spectrophotometer.

#### 2.4.2. Construction of Pseudo-Ternary Phase Diagrams

The pseudo-ternary phase diagrams (PTPDs) were utilized to optimize and determine the stable phase range intended for mixing to create an SMEDDS. The optimal formulation of SMEDDS was indicated by the largest stable microemulsion area, determined by plotting the percentages of oil, water, and surfactant [23]. Various ratios of oil phases, surfactant, and co-surfactant were combined and vortexed to obtain transparent solutions. Then, 500 μL of these mixtures were slowly titrated into 100 mL of water under magnetic stirring (100 rpm) at 37 °C. The formation of the microemulsion (ME) was determined through visual inspection, whereby only transparent or slightly yellowish dispersions with a single-phase transparent fluid system were considered as microemulsions. Conversely, turbid dispersions were classified as coarse emulsions. Based on these observations, the ME region was then delineated on the ternary phase diagram.

#### 2.4.3. Preparation of the BH-SMEDDS

According to the results of the PTPDs, Capmul MCM, Kolliphor RH 40, and 1,2-propanediol were selected as auxiliary materials for BH loading and subsequent characterization. Afterward, BH was dissolved in a mixture of surfactant and co-surfactant (Kolliphor RH 40 and 1,2-propanediol) and the dispersion was shaken at 50 °C for 5 min. Following this, the oil phase (Capmul MCM) was added to the mixture and shaken for another 30 min at 50 °C until the system was stable and uniform. Finally, a clear BH-SMEDDS dispersion was obtained.

### 2.5. Characterization of BH-SMEDDS

The optimal BH-SMEDDS was diluted 50 times with distilled water at 37 °C. The particle size and polydispersity index were determined by dynamic light scattering (DLS, ZetaPlus Analyzer, Brookhaven Instruments Corporation, Nashua, NH, USA) at 25 °C. All samples were measured in triplicates.

The morphology of the BH-SMEDDS and blank SMEDDS was observed by transmission electron microscopy (TEM, HT-7700, Hitachi, Tokyo, Japan). A drop of diluted BH-SMEDDS suspension was deposited on a film-coated copper grid and stained with 2% (*w*/*v*) phosphotungstic acid for 30 s. After being air-dried at room temperature, the morphology of the BH-SMEDDS was observed by TEM. 

To determine the emulsification duration of BH-SMEDDS, 0.5 g of BH-SMEDDS was rapidly injected into 50 mL of distilled water while stirring continuously at 100 rpm/37 °C. The emulsification duration, defined as the time taken until a visibly clear, homogeneous, and emulsified blend was achieved, was evaluated.

### 2.6. Stability Studies of BH-SMEDDS 

To evaluate the stability of BH-SMEDDS after centrifugation, 200 μL of BH-SMEDDS was successively dispersed in 30 mL of deionized water at 37 °C under gentle stirring at 100 rpm. After that, the mixture underwent centrifugation at speeds of 4000, 8000, and 12,000 rpm for 20 min, with observations made to determine if the solution exhibited delamination following centrifugation.

To assess the stability of BH-SMEDDS following dilution, 200 μL of BH SMEDDS was dispersed and diluted 200, 300, and 500 times using varying volumes of deionized water. Subsequently, the resulting mixtures were uniformly emulsified through gentle stirring at 100 rpm at 37 °C. The appearance of the self-emulsified solution was observed, while the self-emulsification time, particle size, and PDI were measured to explore the impact of different dilution times on the stability of BH-SMEDDS.

### 2.7. In Vitro Dissolution Study

The in vitro dissolution study of BH-SMEDDS was performed by the paddle method in accordance with the Chinese Pharmacopeia 2015 using a dissolution tester set at a rotation speed of 100 rpm (UDT-8186, Logan Instruments Corp, Somerset, NJ, USA). To mimic the conditions of the gastrointestinal tract, simulated gastric fluid (pH 1.2) and intestinal fluid (pH 6.8) served as the dissolution media. In brief, 2 mL of samples containing 5 mg of BH were withdrawn from 300 mL of release medium at pre-determined time points of 5, 15, 30, 60, 120, 180, 240, and 300 min with temperature of (37 ± 0.5) °C. Immediately after collection, an equal volume of dissolution medium at the same temperature was replenished. The collected sample solution was filtered through 0.45 µm microporous filter membrane and diluted with methanol. The concentration of BH was determined by UV spectrophotometry. Additionally, the commercially available BH tablets were utilized as the comparative control. The cumulative dissolution percentage of BH in simulated gastric fluid and intestinal fluid was calculated according to Equation (2), and the cumulative dissolution curves were plotted, as follows:

(2) $$\mathrm{Accumulative}\;\mathrm{dissolution}\;\mathrm{percentage}\;\left(\%\right)=\left[\frac{C_{n}\times V+(C_{1}+C_{2}+\cdots +C_{n-1})\times V_{1}}{m}\right]$$

Among them, C_n_ is the concentration of the sample taken at each time point; V is the volume of dissolution medium and V_1_ is the volume of the sample taken at each time point; and m is the amount of drug in the self-nanoemulsion.

### 2.8. In Vitro Cytotoxicity Study

The cytotoxicity of BH-SMEDDS to Caco-2 cells was assessed using the cell counting kit-8 (CCK-8) assay [24]. Initially, Caco-2 cells were seeded on a 96-well cell plate and incubated at 37 °C with 5% CO_2_ for 24 h. Following this, the complete medium was replaced with medium containing different concentrations of BH-SMEDDS (50, 100, 200, 400, 600 μg/mL) or blank SMEDDS (equivalent to the same concentration of BH-SMEDDS). Cells without any treatment were served as the negative control group, while the culture medium was used as the blank control group. After incubating for 24 h, 10 μL of CCK8 solution was added to each well and incubated for an additional 4 h at 37 °C. The absorbance of each well was then measured at 450 nm using a BioTek microplate reader (BioTek Instruments, Inc., Winooski, VT, USA). Cell viability was calculated using the following Formula (3):

(3) $$Cellviability\left(\%\right)=\frac{A_{sample}-A_{blank}}{A_{control}-A_{blank}}\times 100\%$$

where A_sample_ and A_control_ are the mean absorbance value of tested groups and control groups, respectively, and A_blank_ is the absorbance value of the blank medium.

### 2.9. In Vivo Pharmacokinetic Studies

Twelve healthy male SD rats of SPF-grade were randomly divided into two groups and underwent a 12 h fasting period with free access to water prior to the experiment. Commercially available BH tablets were employed as the control formulation in this study. The rats received oral administrations of BH-SMEDDS or BH tablets, each at a BH dosage of 100 mg/kg. An aliquot of 300–500 μL of blood was drawn from the ophthalmic veins of the rats at 5, 15, 30, and 45 min, as well as 1, 2, 3, 4, 6, 8, 12, and 24 h after oral administration. Blood samples were collected in sodium heparin anticoagulant tubes and subsequently centrifuged at 4000 rpm for 10 min. The resulting supernatant plasma was harvested and stored at −20 °C for further analysis. 

To determine the concentration of BH in the plasma samples, 200 μL of each of the samples was transferred to a centrifugal tube and mixed with 600 μL of methanol. After vortexing for 5 min and centrifuging at 7000 rpm for 10 min, the supernatant was collected and dried under nitrogen at 37 °C. Subsequently, the residue was dissolved in 100 μL methanol. The resulting solution was vortexed for 5 min and centrifuged at 12,000 rpm for 5 min, and 20 μL of the supernatant was analyzed by HPLC system (SIL-2A, Agilent Technologies, Santa Clara, CA, USA) at a wavelength of 345 nm. The mobile phase comprised acetonitrile: potassium dihydrogen phosphate (0.033 mol/mL) (33.3:66.6, *v*/*v*) at a flow rate of 1.0 mL/min. The chromatographic separations were performed on an ODS C18 column (4.6 mm × 150 mm, 5 μm) and the column was maintained at 28 °C. Specificity was confirmed by ensuring no interference peak appeared at the retention time of BH when analyzing blank rat plasma spiked with amantadine hydrochloride as the internal standard. Standard solutions for HPLC analysis of BH were prepared by spiking BH stock solutions into blank rat plasma. Following dilution, the concentrations of BH in rat plasma were 0.1, 0.2, 0.5, 1.0, and 1.2 µg/mL, respectively (y = 5.0187X − 0.1972, R^2^ = 0.9990). Validation of the HPLC methods included assessing parameters such as linearity, recovery, and the relative standard deviation of inter-day and intra-day precision (Figures S1–S3, Tables S1 and S2). 

### 2.10. Statistical Analysis

The pharmacokinetic parameters, including the area under the plasma concentration–time curve (AUC), the maximum plasma concentration (C_max_), and the time to reach maximum plasma concentration (T_max_) were calculated by the PK Solver 2.0 software. All experimental data were expressed as the mean ± SD. Statistical analysis was performed using a standard Student’s *t*-test (comparing only two individual groups) in SPSS software (version 27.0) with significance set at *p* < 0.05.

## 3. Results

### 3.1. Equilibrium Solubility and Oil-Water Partition Coefficient of BH

**Figure 1:**
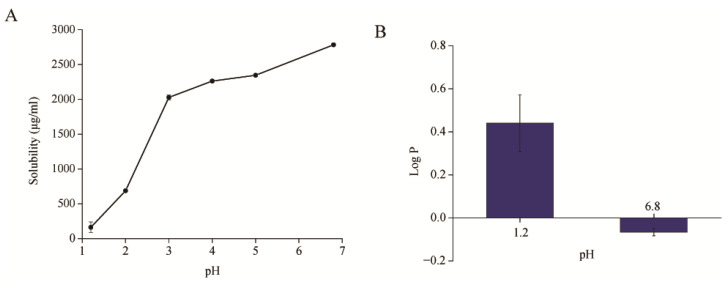
(**A**) Equilibrium solubility of BH in various pH solutions (n = 3); (**B**) the P_o/w_ of BH in 1.2 and 6.8 pH solutions (n = 3).

The equilibrium solubility of BH was assessed in media with varying pH levels. The results revealed significant variability across pH environments, with a general tendency to rise with the increasing pH levels (Figure 1A). Briefly, BH was sparingly soluble at a pH of 1.2 and a pH of 2.0. A notable increase in solubility occurred at a pH of 3.0 (2.03 ± 0.04 mg/mL), followed by a slower increase at a pH of 4.0 and 5.0, with concentrations of 2.26 ± 0.02 mg/mL and 2.34 ± 0.02 mg/mL, respectively. Maximum solubility was observed at a pH of 6.8, reaching 2.78 ± 0.01 mg/mL. It was suggested that BH solubility was consistently lower across all pH conditions, showing a strong pH dependency. Moreover, solubility in the higher pH intestinal environment was significantly greater than in the lower pH gastric environment after oral administration.

The P_o/w_ of BH at a pH of 1.2 and 6.8 are depicted in Figure 1B. It is evident that the P_o/w_ of BH demonstrated a tendency to decrease with increasing medium pH, contrasting with the trend observed in its equilibrium solubility. BH displayed a LogP value ranging between 0 and 1 in a pH 1.2 solution, while at a pH of 6.8, the LogP value dropped below 0, indicating a decrease in BH’s lipophilicity as the pH increases. Typically, a LogP value exceeding 1 suggests high drug lipophilicity, facilitating easy absorption through cell membranes. However, as shown in Figure 1B, BH’s lipophilicity in the gastrointestinal tract is low, corresponding to poor cell membrane permeability. Coupled with its poor solubility at these pH levels, BH is prone to precipitation in the stomach, while its solubility in the intestines increases, though it remains insufficient for enhanced solubility. Consequently, the combination of poor intestinal membrane permeability and low solubility contributes to the low bioavailability of BH and hindered absorption of BH in the gastrointestinal tract. Thus, enhancing the lipophilicity and solubility of BH becomes imperative to address its poor oral bioavailability.

### 3.2. Solubility Studies of BH in Various Excipients

**Figure 2:**
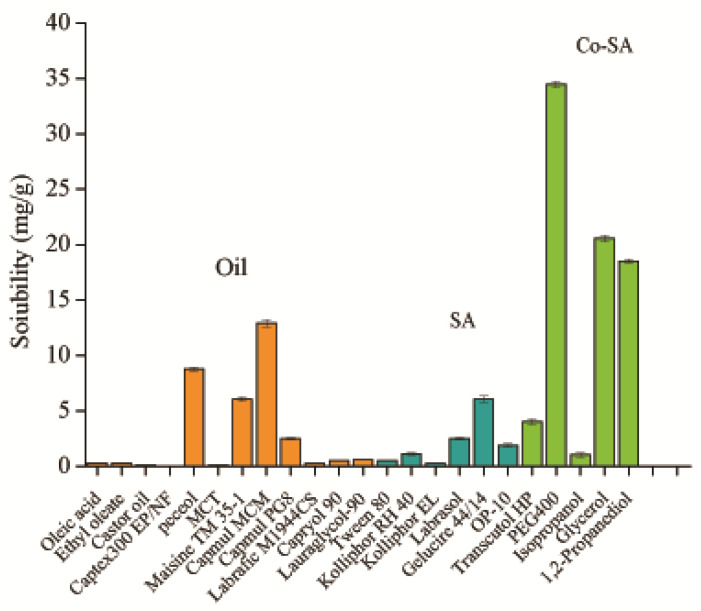
Solubility of BH in common oils, surfactants and co-surfactants (mean ± SD, n = 3). SA: surfactant, Co-SA: co-surfactant.

Insoluble drugs encapsulated within SMEDDS should undergo complete dissolution, and excipients with favorable drug solubility should be prioritized for SMEDDS development. Therefore, various oils, surfactants, and cosurfactants were assessed for their ability to solubilize BH (Figure 2). Initially, oils serve as drug carriers and significantly enhance drug solubility. Notably, Peceol, Maisine TM35-1, Capmul MCM, and Capmul PG8 demonstrated considerable potential in enhancing BH solubility, achieving 8.76 ± 0.20 mg/g, 6.05 ± 0.22 mg/g, 12.95 ± 0.35 mg/g, and 2.49 ± 0.10 mg/g, respectively, compared to less than 2 mg/g in other groups. Furthermore, surfactants diminish the interfacial tension between oil and water, facilitating the formation of minute droplets and enhancing microemulsion stability. Gelucire 44/14, OP-10, and Kolliphor RH40 displayed elevated BH solubility; thus, they were chosen for further investigation. Co-surfactants augment the interface fluidity between oil and water, decreasing interfacial tension and fostering the formation of smaller droplets. Evidently, PEG400, 1,2-propanediol, and glycerol exhibited remarkable solubilization capacity for BH, all surpassing 15 mg/g, hence they were identified as potential cosurfactants for BH-SMEDDS formulation.

Based on the results of the compatibility tests (Table 1), four formulations of the SMEDDS were selected for further pseudo-ternary phase diagram construction. These formulations included Capmul MCM or Capmul PG8 as oil phases, Kolliphor RH 40 as surfactant, and 1,2-propanediol or PEG 400 as co-surfactants.

### 3.3. Pseudo-Ternary Phase Diagrams

**Figure 3:**
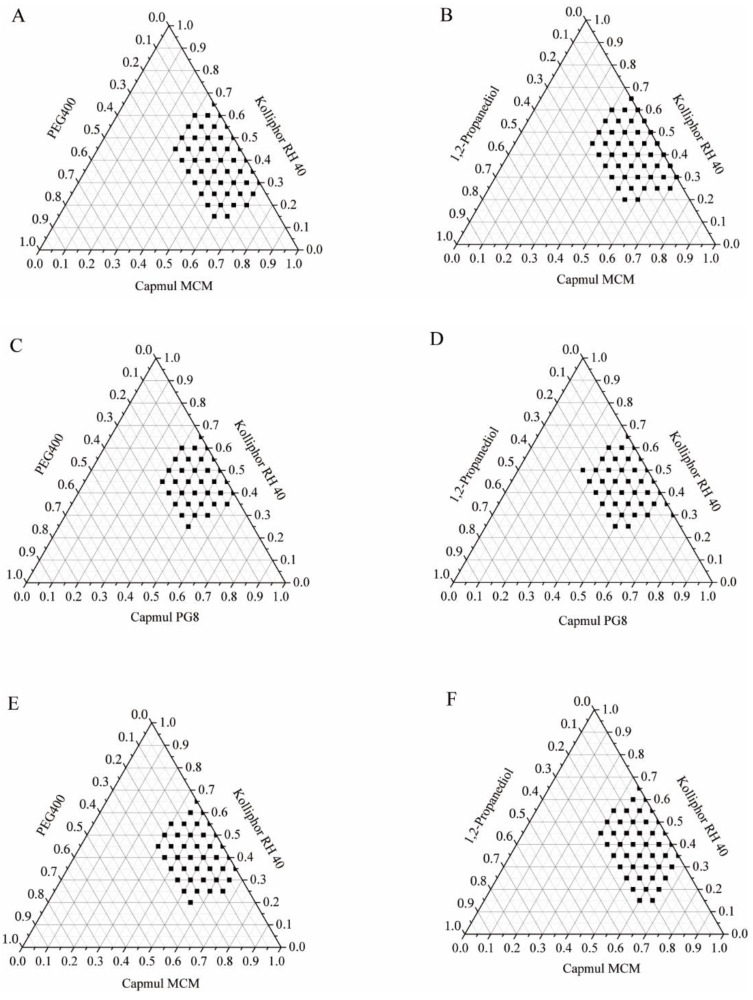
Pseudo-ternary phase diagrams (**A**–**D**) featuring various oils (Capmul MCM or Capmul PG8), a surfactant (Kolliphor RH 40), and co-surfactants (1,2-propanediol or PEG 400) without BH present. (**E**,**F**) Pseudo-ternary phase diagrams composed of Capmul MCM, Kolliphor RH 40, and either PEG 400 or 1,2-propanediol with the loading of BH.

Pseudo-ternary phase diagrams were used to analyze the phase behavior of SMEDDS, which consists of the following three components: oil, surfactant, and co-surfactant, aiding in the identification of the optimal formulation (Figure 3). Following compatibility studies, Kolliphor RH 40, with a hydrophilic–lipophilic balance (HLB) value ranging from 14 to 16, was chosen as the surfactant. Investigations ensued regarding the self-emulsifying capabilities of Kolliphor RH 40 in combination with either 1,2-propanediol or PEG 400. Both 1,2-propanediol (HLB 10.5) and PEG 400 (HLB 12.25) were assessed as co-surfactants. Microemulsion regions are indicated by the shaded areas on the phase diagrams, while the unshaded portions represent the turbid regions. A larger microemulsion area suggests superior self-microemulsifying performance. As observed in Figure 3A,B when Capmul MCM served as the oil, the larger microemulsion region was exhibited; thus, Capmul MCM was selected as the oil. However, no significant difference in self-emulsifying performance was observed between 1,2-propanediol and PEG 400 as co-surfactants in the absence of BH. Conversely, upon incorporating BH into SMEDDS, a larger microemulsion area was noted with 1,2-propanediol as the co-surfactant compared to PEG 400 (Figure 3E,F). This led to the selection of 1,2-propanediol as the co-surfactant. Consequently, the combination of Capmul MCM, Kolliphor RH 40, and 1,2-propanediol was selected for the development of BH-SMEDDS.

### 3.4. Formulation Optimization of BH-SMEDDS

**Figure 4:**
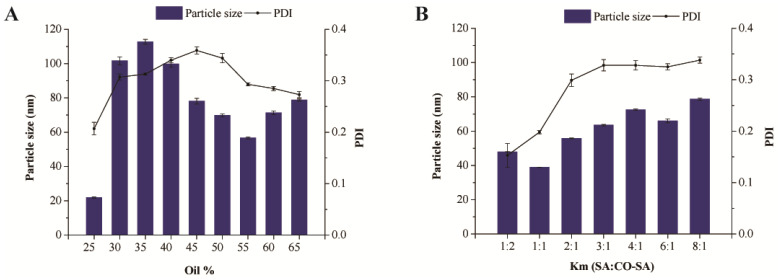
The effect of (**A**) oil concentration and (**B**) Km on the mean particle size and PDI of BH-SMEDDS.

The investigations further delved into the particle size and polydispersity index (PDI) of emulsions, focusing on their significance in determining the optimal ratio of oil, surfactant, and co-surfactant. Figure 4A illustrates a discernible trend wherein the particle size initially decreases and then increases with the increase in the oil phase proportion. At 55% oil phase concentration, the smallest particle size was observed, accompanied by a polydispersity index (PDI) below 0.3. Subsequent increments in the oil phase proportion led to a gradual decrease in PDI. Hence, to ensure both minimal particle size and a PDI below 0.3, the ideal oil phase concentration for self-microemulsion formulation was determined to be 55%. When the concentration of emulsifier is low, there are fewer molecules adsorbed at the interface between the organic and aqueous phases, leading to a weaker liquid interfacial film and an unstable emulsion. As the emulsifier concentration increases to a certain level, the interfacial film forms from closely packed, oriented adsorbed molecules, resulting in a stronger interfacial film and a more stable emulsion. However, if the limonene concentration becomes too high, the droplet size increases due to Ostwald ripening [25]. 

As depicted in Figure 4B, when varying the weight ratio of surfactant to co-surfactant (Km) within the range of 1:2 to 8:1, there was a gradual increase in both the particle size and the PDI of the microemulsion with increasing Km. The smallest particle size of the emulsion was observed at Km 1, measuring (38.9 ± 0.1) nm, indicating a more stable emulsion system post self-microemulsification. Ultimately, it was determined that the optimal proportion of emulsifier in the self-microemulsion formulation was 22.5%, with an equivalent proportion of co-emulsifier at 22.5%. The co-emulsifier can affect the surface activity and HLB of the emulsifier. An excessive or insufficient amount of co-emulsifier will impact the formation of the droplets [17]. Therefore, the optimal formulation for BH-SMEDDS was determined as 55% oil phase (Capmul MCM), 22.5% surfactant (Kolliphor RH 40), and 22.5% co-surfactant (1,2-propanediol).

The drug content also affects the stability of the droplets. If the dosage is excessive, drug leakage may occur. As indicated in Table 2, drug precipitation was observed with a BH dosage of 22 mg/g after 48 h of storage at room temperature. At BH dosages equal to or below 20 mg/g, no drug precipitation was observed within the microemulsion. Hence, it was concluded that the optimal BH dosage should be set at 20 mg/g.

### 3.5. Characterization of BH-SMEDDS

**Figure 5:**
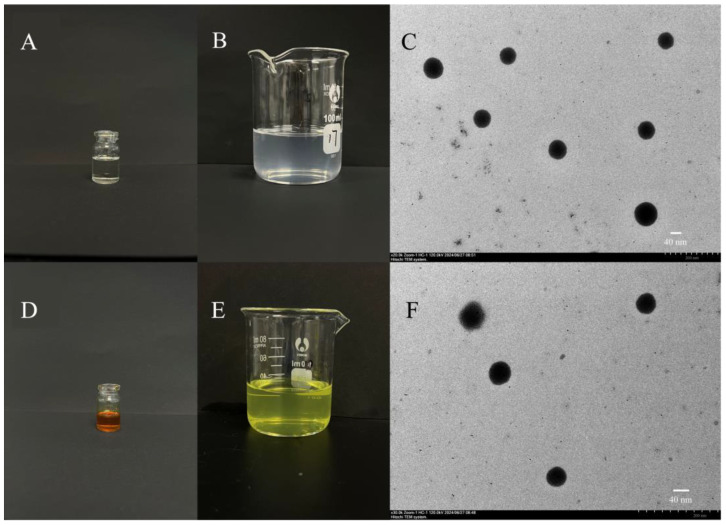
(**A**) Appearance of blank SMEDDS and (**B**) microemulsion after self-microemulsifying. (**C**) TEM image of blank SMEDDS (scale bar = 40 nm). (**D**) Appearance of BH-SMEDDS and (**E**) microemulsion after self-microemulsifying. (**F**) TEM image of BH-SMEDDS (scale bar = 40 nm).

The blank SMEDDS and BH-SMEDDS solutions both remained clear and transparent. The blank SMEDDS displayed a faint blue glow; meanwhile, the BH-SMEDDS, due to the yellow hue of BH, exhibited a slightly milky, yellowish light post self-microemulsion (Figure 5B,E). The average particle sizes of blank SMEDDS and BH-SMEDDS were 38.9 ± 0.10 nm and 47.2 ± 0.10 nm, respectively, with polydispersity indexes (PDI) of 0.198 ± 0.003 and 0.262 ± 0.006. Figure 5C,F illustrate the morphology of blank SMEDDS and BH-SMEDDS, showing uniformly spherical microemulsion droplets of approximately 50 nm. Additionally, the emulsification time in water for blank SMEDDS and BH-SMEDDS was examined. Previous studies have indicated that a duration of 2 min serves as a benchmark for assessing the emulsification process [26]. It was also suggested that a moderate stirring speed would quickly mix the oil and water (with the appropriate ratio), leading to effective emulsification and rapid completion of the process [27]. In our study, the emulsification time in water for blank SMEDDS and BH-SMEDDS was recorded as 20.37 ± 0.23 s and 26.02 ± 0.24 s, respectively, indicating efficient emulsification and suggesting that the incorporation of BH did not notably affect the emulsification duration. 

### 3.6. Stability Studies

As indicated in Table 3, there were no notable alterations in particle size or visual characteristics of BH-SMEDDS even after dilution ranging from 200 to 500 times. It maintained its pale yellow, transparent liquid state without any signs of cloudiness or separation. These findings suggest that the formulated BH-SMEDDS exhibits favorable stability.

### 3.7. In Vitro Dissolution Studies

**Figure 6:**
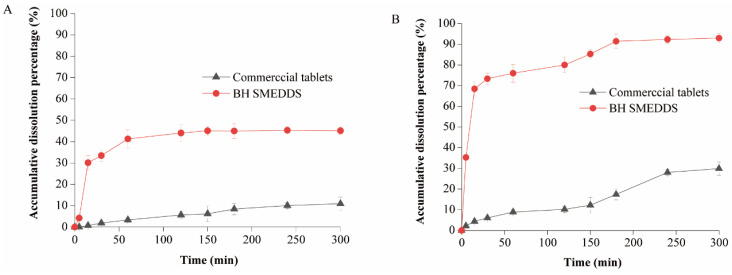
The dissolution curves of the BH-SMEDDS and commercial tablets in (**A**) the simulated gastric fluid (pH = 1.2, n = 3), and (**B**) the simulated intestinal fluid (pH = 6.8, n = 3).

The in vitro release of BH-SMEDDS was assessed in simulated gastric fluid and intestinal fluid. As shown in Figure 6A, in the simulated gastric fluid (pH 1.2), BH-SMEDDS exhibited rapid release within the initial 60 min, followed by a stable release, ultimately reaching only 45.1 ± 1.7% release of BH within 300 min. Conversely, in the simulated intestinal fluid, the release of BH-SMEDDS showed considerable enhancement, with approximately 70% drug release observed within the first 15 min, and eventually reaching 93.1 ± 2.3% release within 300 min. When compared to commercially available tablets, BH-SMEDDS demonstrated significantly higher dissolution rates in both simulated gastric and intestinal fluids. This result suggests that the formulation of BH as a self-microemulsion markedly improved drug dissolution in simulated digestive fluids. This improvement aligns with the trend observed in the commercially available formulation in both simulated gastric and intestinal fluids. This enhanced dissolution in simulated intestinal fluid is likely attributed to the better solubility of BH after incorporating in SMEDDS under neutral or alkaline pH conditions compared to acidic pH, facilitating effective dissolution and promoting BH dissolution. 

Additionally, BH-SMEDDS and commercial BH tablets containing 5 mg of BH were used for the dissolution study. Given that BH had a solubility of 162 μg/mL at a pH of 1.2, a release medium volume of 300 mL was used, which was more than three times the required volume (92.6 mL) for maintaining sink conditions in the in vitro dissolution experiment. Likewise, with the solubility of BH of 2.78 mg/mL at a pH of 6.8, the volume of release medium also met the sink condition requirements for the in vitro dissolution study.

### 3.8. Cytotoxicity Studies of BH-SMEDDS

**Figure 7:**
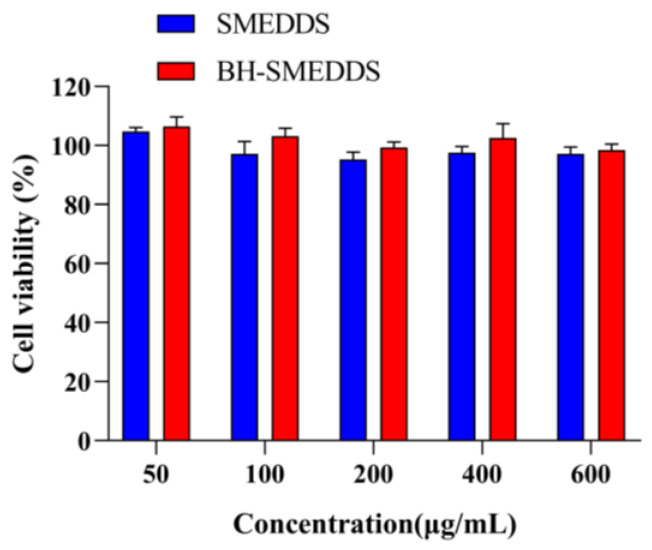
The cytotoxicity of blank SMEDDS and BH-SMEDDS at the concentration of 50 to 600 µg/mL (mean ± SD, n = 3).

Owing to the elevated concentration of surfactants utilized in SMEDDS, in vitro cell toxicity has been reported in many published studies [28,29]. Hence, it is imperative to assess the cytotoxicity of both blank SMEDDS and BH-SMEDDS. As shown in Figure 7, neither blank SMEDDS nor BH-SMEDDS demonstrated notable cytotoxicity within the dosage range of 50 to 600 μg/mL, as determined by the CCK-8 assay. This suggests that the formulated microemulsion exhibits minimal cytotoxic effects on the Caco-2 cells. Furthermore, the cell viability (%) for both blank SMEDDS and BH-SMEDDS remained above 95%. Therefore, BH-SMEDDS demonstrates substantial safety with minimal impact on the gastrointestinal membrane, making it a promising formulation for potential clinical application.

### 3.9. In Vivo Pharmacokinetic Study of BH-SMEDDS 

**Figure 8:**
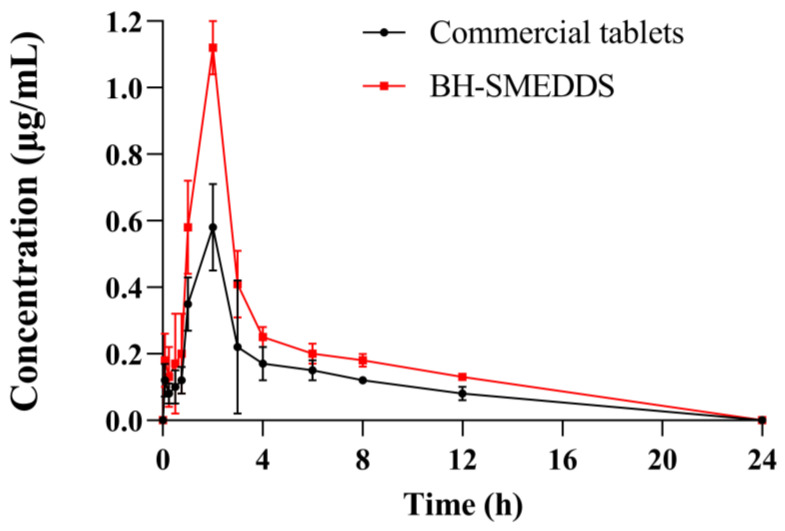
Plasma concentration–time profiles of BH after oral administration of the BH-SMEDDS and commercial tablets in rats at a dose of 100 mg/kg (mean ± SD, n = 6).

The plasma concentration–time profiles of BH following oral administration of BH-SMEDDS and the reference preparation are depicted in Figure 8, and the corresponding pharmacokinetic parameters are summarized in Table 4. As illustrated in the figure, the control group (commercial tablets) reached the maximum plasma concentration (C_max_) of 0.55 ± 0.06 µg/mL at 2 h after oral administration. In contrast, the BH-SMEDDS group achieved a C_max_ 209.1% significantly higher than the BH tablets, reaching 1.15 ± 0.05 µg/mL at the same time point (*p* < 0.05). Moreover, the area under the curve (AUC_0–∞_) value for BH-SMEDDS was 5.116 ± 0.829 µg∙h/mL, which was 1.63-fold greater than that of the commercial tablets (3.138 ± 0.263 µg∙h/mL) with *p* values less than 0.01. The significantly elevated C_max_, faster rate of reaching C_max_, and notably increased AUC_0–∞_ value collectively suggest the enhanced absorption of BH with BH-SMEDDS. In terms of elimination half-time t_1/2_, BH-SMEDDS exhibited prolonged elimination compared to the commercial tablets, with t_1/2_ increased from 5.021 ± 0.614 to 9.347 ± 0.532 h. The relative bioavailability of the BH-SMEDDS to the marketed preparation was 163.03%. In previous studies on BH nanoemulsion, the C_max_ of BH nanoemulsion was 1.56-fold higher than that of control group (BH suspension) with the highest concentration reached at 4.7 h after administration [11]. In comparison, BH-SMEDDS demonstrated a 2.09-fold higher C_max_ within 2 h, indicating faster oral absorption and onset of action. This may be due to the enhanced dissolution of BH-SMEDDS in the GI tract. 

In conclusion, BH-SMEDDS could effectively improve the oral absorption and bioavailability of BH after administration; thus, BH-SMEDDS would provide great potential for the drug applications in clinical settings. 

BH is a BCS class III drug, characterized by high solubility and low permeability [30]. Its solubility is highly pH-dependent, and free BH tends to precipitate and aggregate in the acidic environment of the gastrointestinal tract, leading to poor solubility and limited absorption. The low permeability of BH is attributed to its low P_o/w_ values and lipophilicity. Thus, enhancing BH solubility and permeability is crucial for improving its bioavailability. In pharmacokinetic studies, as the circulating blood volume of SD rats typically ranges from 15 to 20 mL, the blood sample volume withdrawn at each time point in this study is 300–500 μL, amounting to 15–20% of the total circulating blood volume within 24 hours. This approach aligns with the published articles and guidelines for blood collection in laboratory animals [31,32,33]. However, withdrawing large volumes of blood exceeding 25% of circulating blood volume can lead to pharmacokinetic alterations and may influence the calculation of the drug’s half-life. Although replenishing blood volume after sampling can partially mitigate excessive blood loss, it cannot entirely eliminate the impact of substantial blood loss.

In this study, BH-SMEDDS was developed using oil phase, surfactant, and co-surfactant. The surfactant and co-surfactant reduce the interfacial surface tension, improving membrane permeability, and facilitating absorption through the membrane. Additionally, BH was incorporated into the oil phase of SMEDDS, forming fine oil-in-water droplets (<50 nm). The increased surface area and smaller droplets enhance drug transportation across the gastrointestinal membrane, significantly improving absorption. The enhanced permeability, increased drug transportation, and improved solubility of BH as observed in in vitro dissolution studies, collectively contribute to the superior oral bioavailability of our BH-SMEDDS formulation. In the future, preclinical studies including pharmacodynamics research, investigation of the absorption mechanism of BH-SMEDDS, and toxicology studies (such as acute toxicity testing) will be necessary to better understand the potential of BH-SMEDDS. Following these preclinical evaluations, clinical trials involving humans can then be conducted.

## 4. Conclusions

In this study, the BH-SMEDDS formulation containing Capmul MCM, Kolliphor RH 40, and 1,2-propanediol was successfully developed to enhance the oral delivery of BH in rats. The characterization results indicated that BH-SMEDDS formed stable and uniform spheres with a narrow droplet size distribution. The in vitro release studies demonstrated a significant improvement in BH dissolution, particularly in simulated intestinal fluid. Moreover, in vitro cytotoxicity assessments showed that BH-SMEDDS was safe, exerting minimal impact on the gastrointestinal membrane. In addition, the in vivo pharmacokinetic investigations revealed an enhanced bioavailability of BH-SMEDDS compared to commercial tablets. Overall, our findings suggest that SMEDDS could represent a promising formulation for improving oral absorption and bioavailability of the BCS class III drug, BH. This may pave the way for future clinical research and applications of BH.

## Figures and Tables

**Table 1:** Compatibility test of single oil phase, surfactant, and co-surfactant.

Compatibility	Self-Emulsifying Time	Appearance	Nanoemulsion Formation
Peceol/Kolliphor RH 40/PEG400	<1 min	D	No
Peceol/Kolliphor RH 40/Glycerin	>1 min	E	No
Peceol/Kolliphor RH 40/1,2-propanediol	<1 min	D	No
Peceol/Labrasol/PEG400	>1 min	E	No
Peceol/Labrasol/Glycerin	<1 min	D	No
Peceol/Labrasol/1,2-propanediol	>1 min	E	No
Peceol/Gelucire 44/14/PEG400	>1 min	E	No
Peceol/Gelucire 44/14/Glycerin	<1 min	D	No
Peceol/Gelucire 44/14/1,2-propanediol	<1 min	D	No
Peceol/OP-14/PEG400	<1 min	C	No
Peceol/OP-14/Glycerin	<1 min	D	No
Peceol/OP-14/1,2-propanediol	<1 min	C	No
Maisine CC/Kolliphor RH 40/PEG400	>1 min	E	No
Maisine CC/Kolliphor RH 40/Glycerin	<1 min	D	Yes
Maisine CC/Kolliphor RH 40/1,2-propanediol	>1 min	E	No
Maisine CC/Labrasol/PEG400	>1 min	E	No
Maisine CC/Labrasol/Glycerin	>1 min	E	No
Maisine CC/Labrasol/1,2-propanediol	>1 min	E	No
Maisine CC/Gelucire 44/14/PEG400	>1 min	E	No
Maisine CC/Gelucire 44/14/Glycerin	>1 min	E	No
Maisine CC/Gelucire 44/14/1,2-propanediol	>1 min	E	No
Maisine CC/OP-14/PEG400	<1 min	D	No
Maisine CC/OP-14/Glycerin	<1 min	D	No
Maisine CC/OP-14/1,2-propanediol	<1 min	D	No
Capmul MCM/Kolliphor RH 40/PEG400	<1 min	A	Yes
Capmul MCM/Kolliphor RH 40/Glycerin	<1 min	E	No
Capmul MCM/Kolliphor RH 40/1,2-propanediol	<1 min	A	Yes
Capmul MCM/Labrasol/PEG400	>1 min	E	No
Capmul MCM/Labrasol/Glycerin	>1 min	E	No
Capmul MCM/Labrasol/1,2-propanediol	>1 min	E	No
Capmul MCM/Gelucire 44/14/PEG400	<1 min	C	No
Capmul MCM/Gelucire 44/14/Glycerin	<1 min	D	No
Capmul MCM/Gelucire 44/14/1,2-propanediol	<1 min	D	No
Capmul MCM/OP-14/PEG400	<1 min	D	No
Capmul MCM/Gelucire 44/14/Glycerin	<1 min	D	No
Capmul MCM/Gelucire 44/14/1,2-propanediol	<1 min	D	No
Capmul PG8/Kolliphor RH 40/PEG400	<1 min	A	Yes
Capmul PG8/Kolliphor RH 40/Glycerin	<1 min	E	No
Capmul PG8/Kolliphor RH 40/1,2-propanediol	<1 min	A	Yes
Capmul PG8/Labrasol/PEG400	>1 min	E	No
Capmul PG8/Labrasol/Glycerin	>1 min	E	No
Capmul PG8/Labrasol/1,2-propanediol	<1 min	D	No
Capmul PG8/Gelucire 44/14/PEG400	<1 min	C	No
Capmul PG8/Gelucire 44/14/Glycerin	<1 min	D	No
Capmul PG8/Gelucire 44/14/1,2-propanediol	<1 min	D	No
Capmul PG8/OP-14/PEG400	<1 min	C	No
Capmul PG8/OP-14/Glycerin	>1 min	E	No
Capmul PG8/OP-14/1,2-propanediol	<1 min	D	No

**Table 2:** The effects of drug loading of BH on the stability and particle size (n = 3).

Drug Content (mg/g)	Crystallization	Particle Size (nm)	PDI	Dissociation after Centrifugation
14	No	54.5 ± 0.4	0.240 ± 0.003	No
16	No	48.0 ± 0.5	0.257 ± 0.008	No
18	No	49.9 ± 0.2	0.257 ± 0.014	No
20	No	50.0 ± 0.4	0.262 ± 0.006	No
22	Yes	37.2 ± 0.2	0.110 ± 0.090	Yes

**Table 3:** The effects of different dilution ratios on BH-SMEDDS (n = 3).

Dilution Ratio	Self-Emulsifying Time (s)	Particle Size (nm)	PDI	Appearance
200	22.00 ± 0.63	42.3 ± 0.4	0.113 ± 0.048	Translucent/light yellow opalescence
300	20.04 ± 0.21	43.0 ± 0.2	0.123 ± 0.023	Translucent/light yellow opalescence
500	18.09 ± 0.37	44.8 ± 1.2	0.133 ± 0.064	Translucent/light yellow opalescence

**Table 4:** Pharmacokinetic parameters after oral administration of the BH-SMEDDS and commercial tablets in rats (n = 6).

Parameters	Commercial Tablets	BH SMEDDS
C_max_ (µg/mL)	0.55 ± 0.06	1.15 ± 0.05 *
T_max_ (h)	2.000 ± 0.000	2.000 ± 0.000
t_1/2_ (h)	5.021 ± 0.614	9.347 ± 0.532
AUC_0–t_ (µg/mL·h)	2.278 ± 0.282	3.114 ± 0.247 *
AUC_0–∞_ (µg/mL·h)	3.138 ± 0.263	5.116 ± 0.829 **
Relative bioavailability (%)	-	163.03

* *p* < 0.05, ** *p* < 0.01 versus the commercial tablets.

## Data Availability

The original contributions presented in this study are included in the article/Supplementary Materials, further inquiries can be directed to the corresponding author/s.

## Supplementary Materials

The following supporting information can be downloaded at: https://www.mdpi.com/article/10.3390/pharmaceutics16091116/s1, Figure S1: HPLC chromatograms of blank plasma (A); blank plasma sample with palmatine chloride (B); blank plasma sample with BH (C); blank plasma sample with BH and palmatine chloride (D). Concentration of BH and palmatine chloride was 0. 5 μg/mL and 0.2 μg/mL; Figure S2: Standard curve of BH in rat plasma; Figure S3: (A) Limit of detection (LOD) and (B) limit of quantitation (LOQ) (10 ng/mL and 50 ng/mL); Figure S4: (A) The cytotoxicity of blank SMEDDS and BH-SMEDDS after 48 h incubation, (B) The cytotoxicity of 10 % DMSO after 24 h and 48 h incubation; Table S1: The precision of BH determination in pharmacokinetic studies (n = 3); Table S2: The recovery of BH determination in pharmacokinetic studies (n = 3).
