# Supplementary material for: Self-Microemulsifying Drug Delivery System to Enhance Oral Bioavailability of Berberine Hydrochloride in Rats
Source: Pharmaceutics. 2024 Aug 24;16(9):1116. doi: 10.3390/pharmaceutics16091116 (PMC11435259; doi:10.3390/pharmaceutics16091116)
Supplement: Supplementary file 1 [file pharmaceutics-16-01116-s001.zip › pharmaceutics-3143095-supplementary.pdf]

Self-Microemulsifying Drug Delivery System to Enhance Oral Bioavailability of Berberine Hydrochloride in Rats

Xiaolan Chen [1,†], Haifeng Yang [1,†], Longyu Shi [2], Yujuan Mao [1], Lin Niu [1], Jing Wang [1], Haifeng Chen [1], Jiping Jia [1], Jingxuan Wang [1], Jiajie Xue [1], Yan Shen [3], Chunli Zheng [3], Yu Tian [4,5,*] and Yi Zheng [1,*]

[1] Department of Pharmaceutics, Jiangsu Agri-Animal Husbandry Vocational College, No.8, Fenghuang East Road, Taizhou 225300, China

[2] College of Life Sciences, China Pharmaceutical University, Nanjing 210009, China

[3] Department of Pharmaceutics, China Pharmaceutical University, Nanjing 210009, China

[4] School of Medicine, Shanghai University, Shanghai 200444, China

[5] Institute of Geriatrics (Shanghai University), Affiliated Nantong Hospital of Shanghai University (The Sixth People's Hospital of Nantong), School of Medicine, Shanghai University, Nantong 201613, China

[†] These authors contributed equally to this work.

[*] **Corresponding author:**

Name: Yu Tian

Address: School of Medicine, Shanghai University, Shanghai 200444, China

Email: shu_yutian@shu.edu.cn

Name: Yi Zheng

Address: Jiangsu Agri-animal Husbandry and Veterinary College, Taizhou, Jiangsu Province 225300, China

Tel: 0086-523-86356166

Fax: 0086-523-8615999.

Email: 1997010030@jsahvc.edu.cn

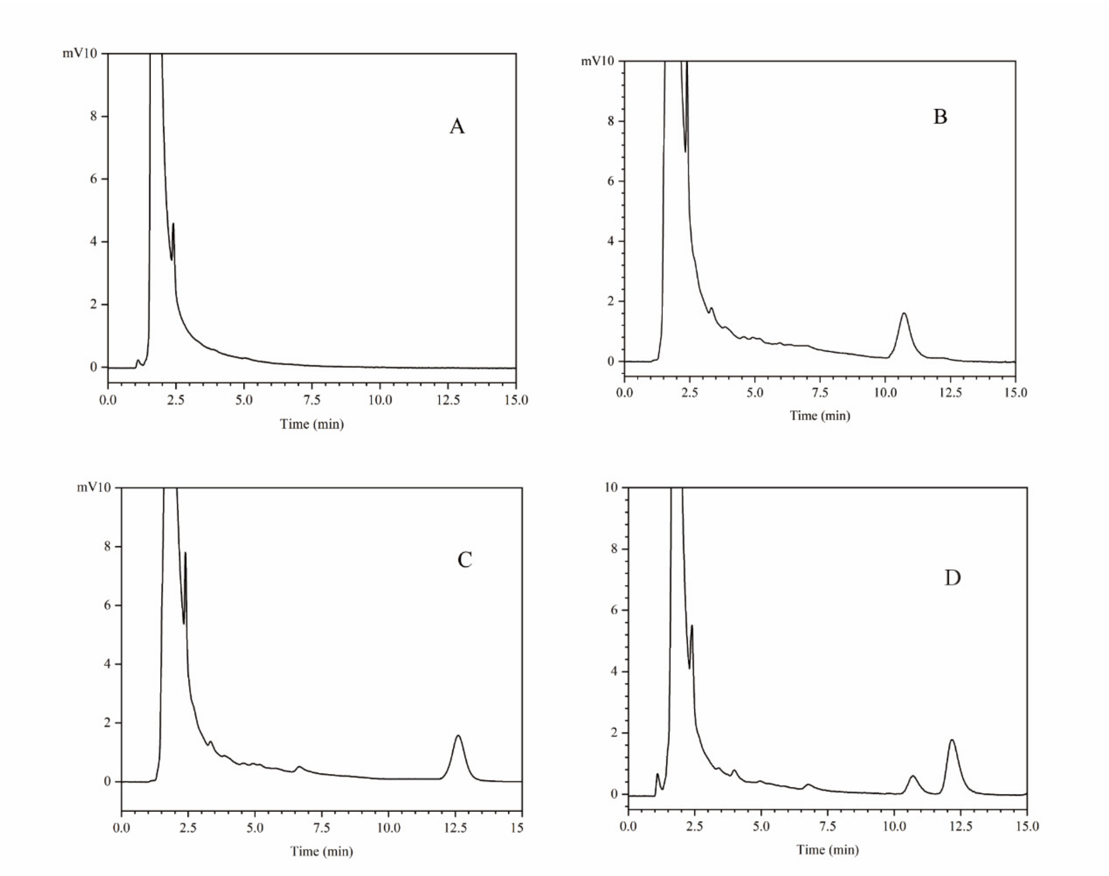

Figure S1. HPLC chromatograms of blank plasma (A); blank plasma sample with palmatine chloride (B); blank plasma sample with BH (C); blank plasma sample with BH and palmatine chloride (D). Concentration of BH and palmatine chloride was 0. 5 μg/mL and 0.2 μg/mL.

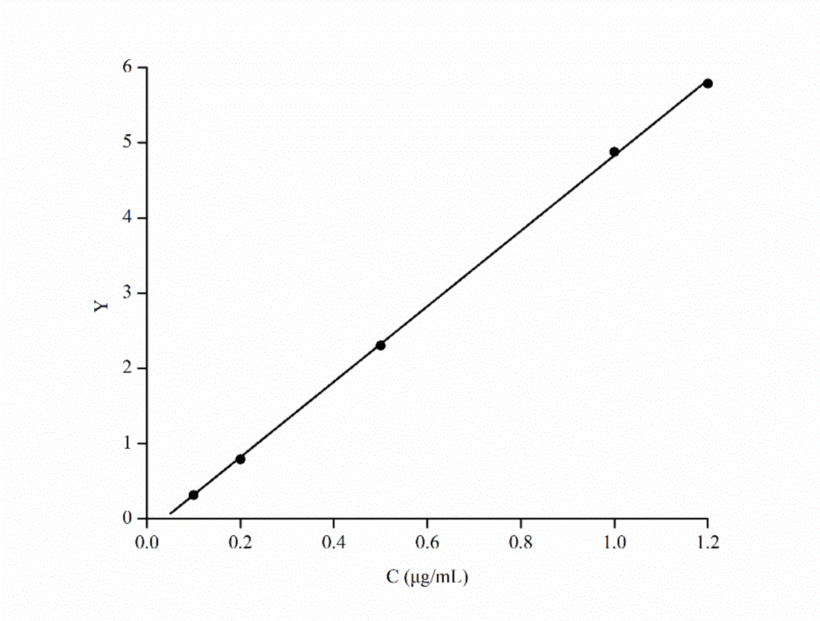

Figure S2. Standard curve of BH in rat plasma

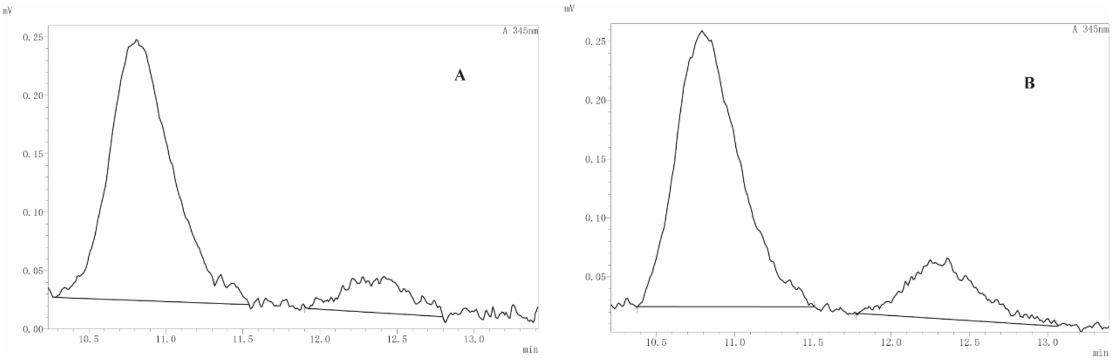

Figure S3. (A) Limit of detection (LOD) and (B) limit of quantitation (LOQ) (10 ng/mL and 50 ng/mL).

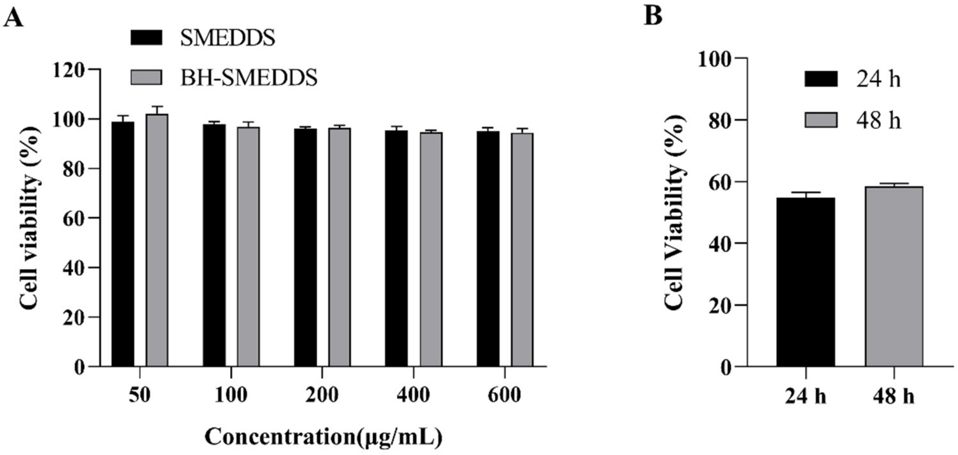

Figure S4. (A) The cytotoxicity of blank SMEDDS and BH-SMEDDS after 48 h incubation, (B) The cytotoxicity of 10 % DMSO after 24 h and 48 h incubation.

Table S1. The precision of BH determination in pharmacokinetic studies (n=3)

	Concentration (μg/mL)	Mean±SD (μg/mL)	RSD (%)
Intra-day	0.05	0.051±0.002	3.01
	0.2	0.199±0.007	2.36
	1.2	1.202±0.004	0.36
Inter-day	0.05	0.049±0.001	2.32
	0.2	0.200±0.005	2.46
	1.2	1.202±0.007	0.58

Table S2. The recovery of BH determination in pharmacokinetic studies (n=3)

Added (μg/mL)	Measured (μg/mL)	Recovery (%)	Mean±SD (%)	RSD (%)
0.1	0.104	104.00	100.33±3.21	3.20
	0.099	99.00		

	0.098	98.00		
	0.509	102.40		
0.5	0.512	101.80	101.13±1.70	1.68
	0.496	99.20		
	1.205	100.42		
1	1.204	100.33	100.00±0.65	0.65
	1.191	99.25		
